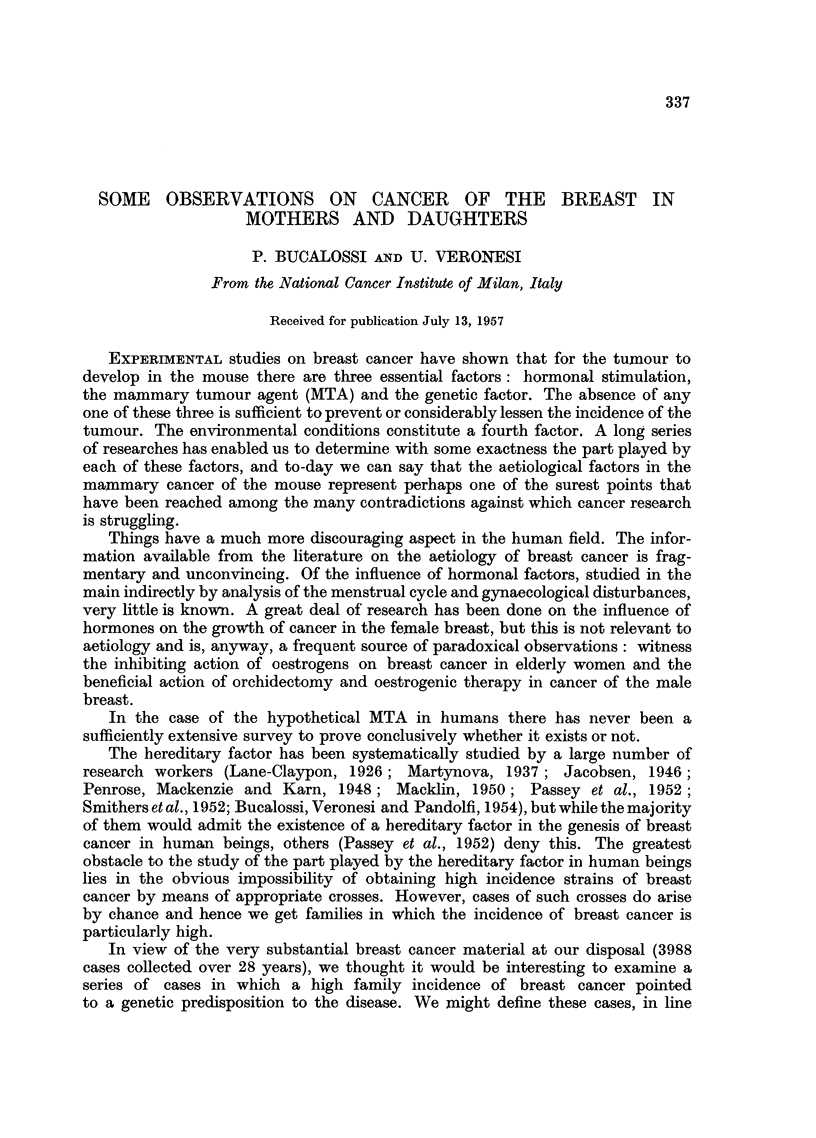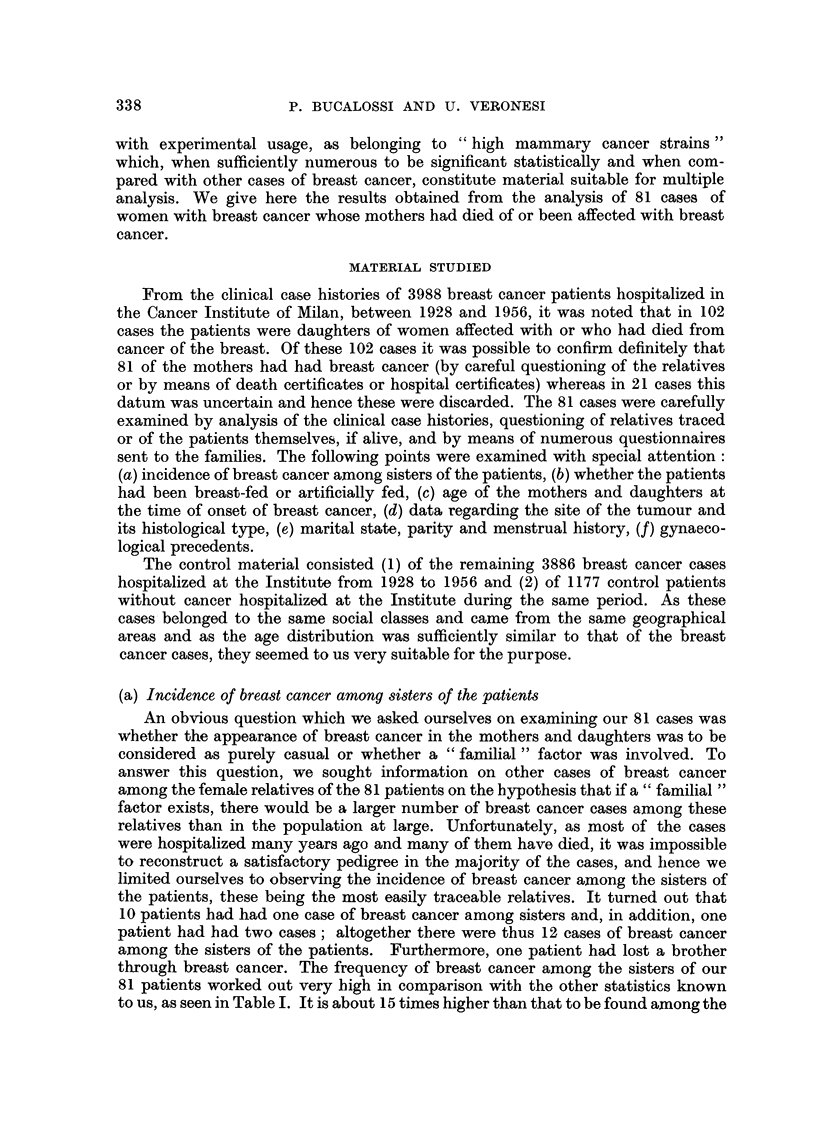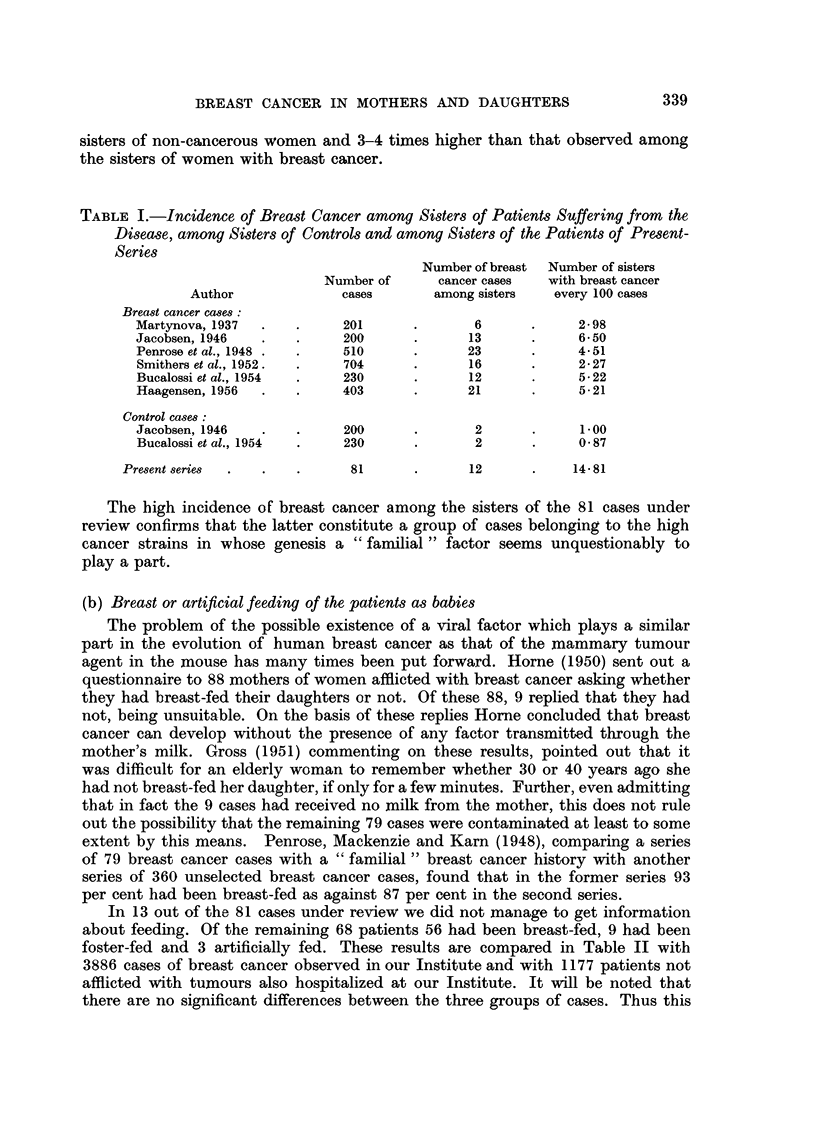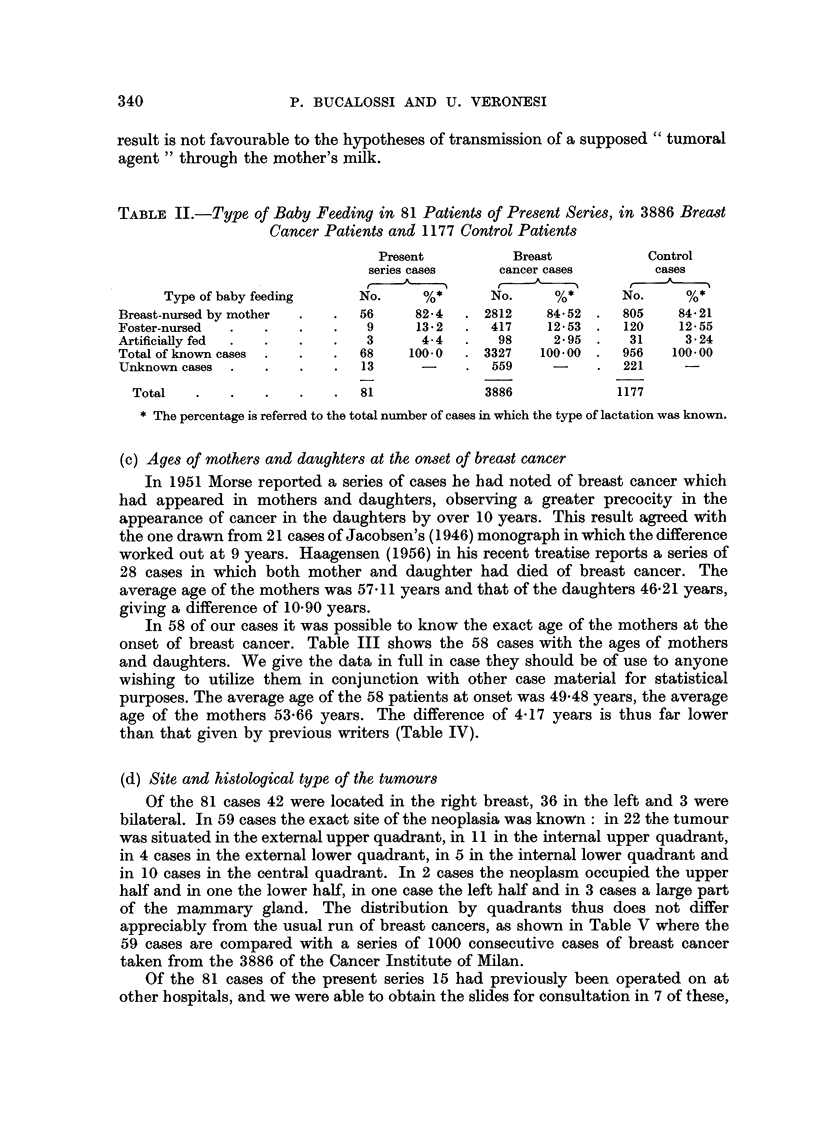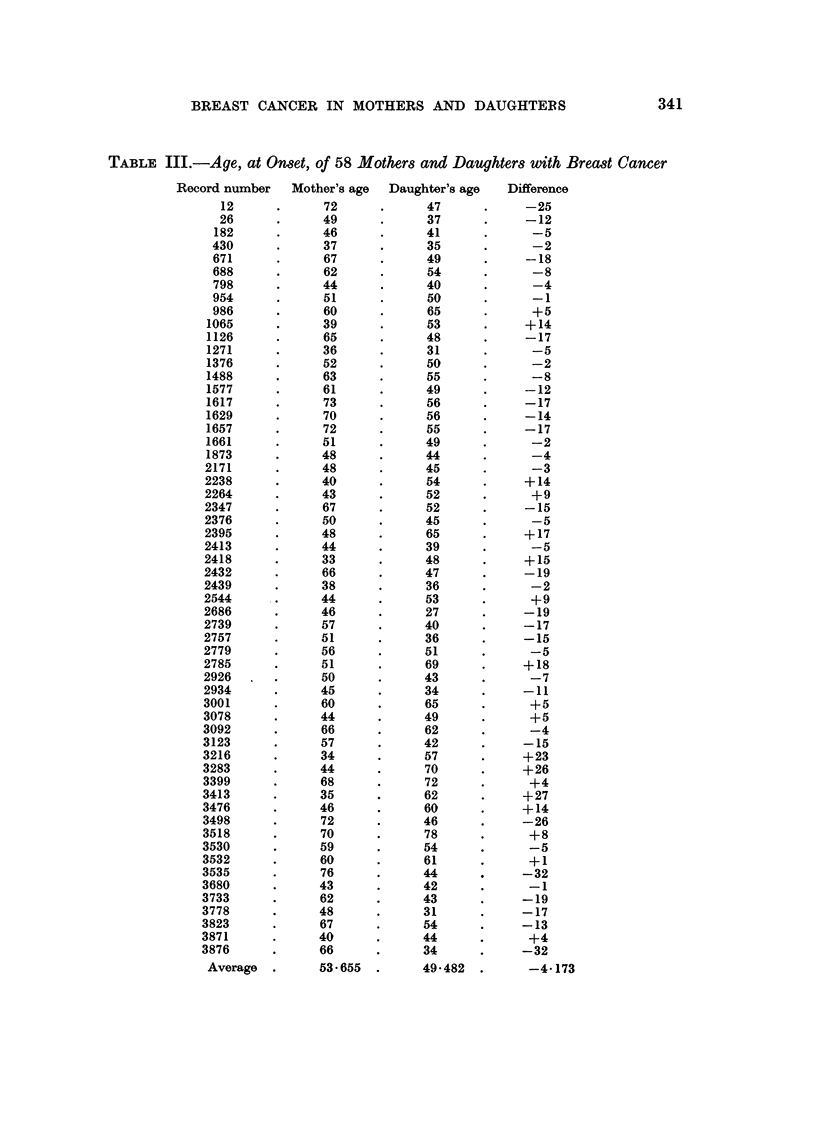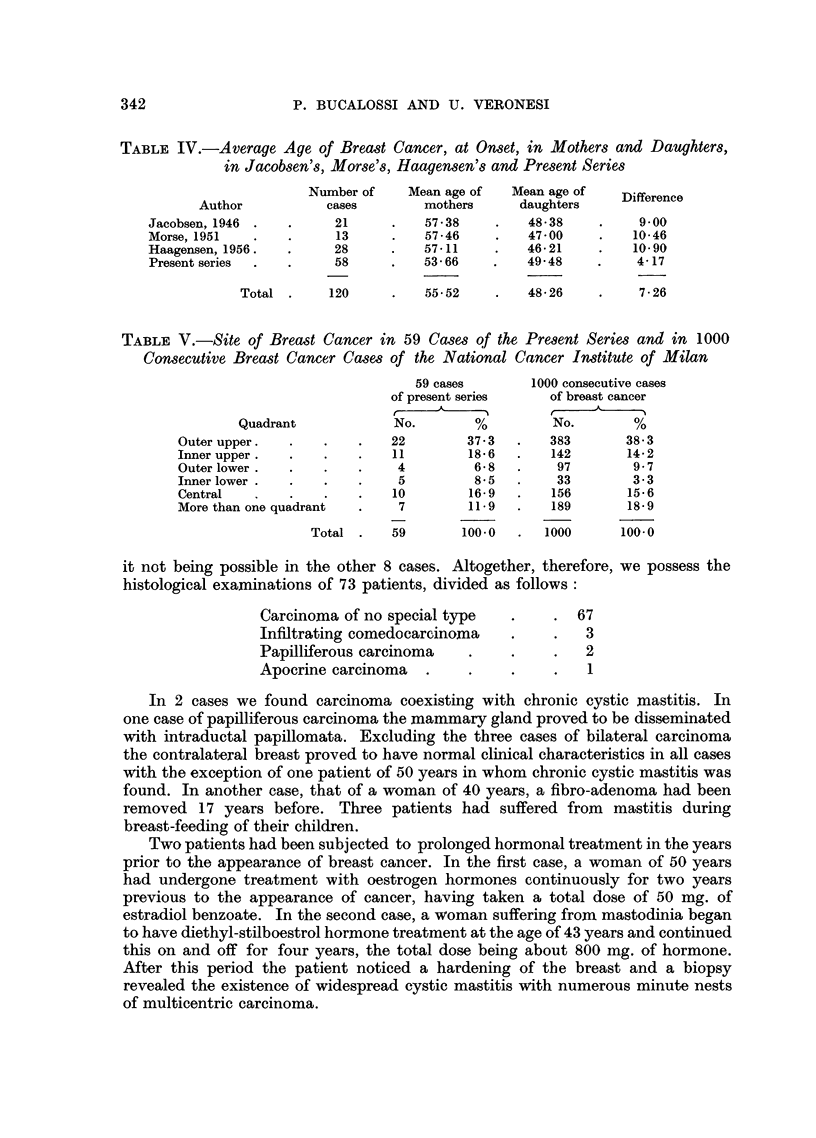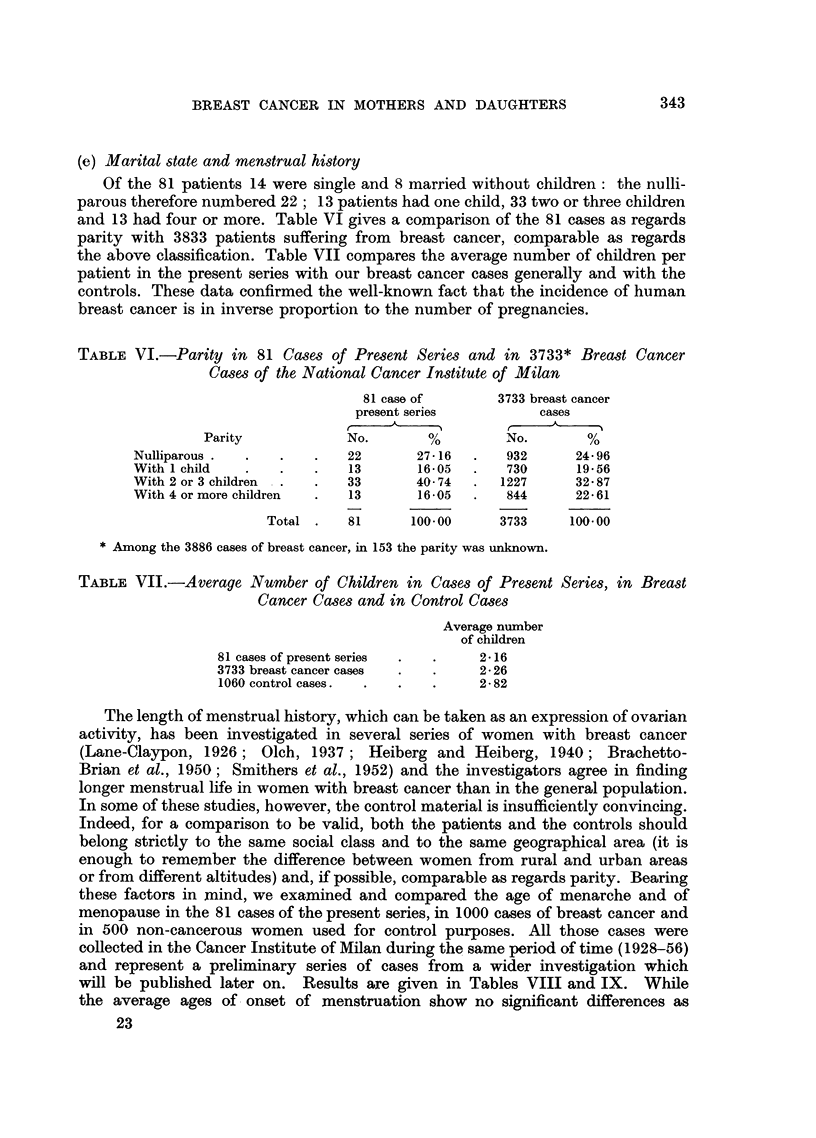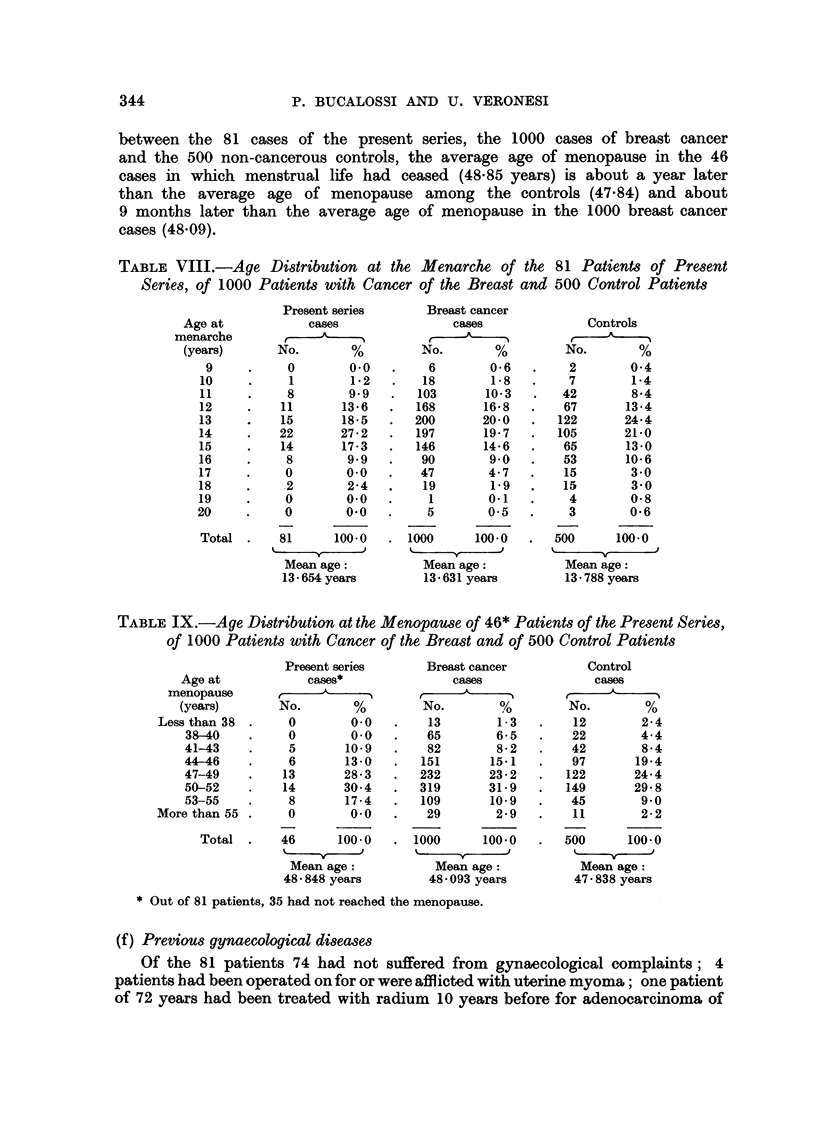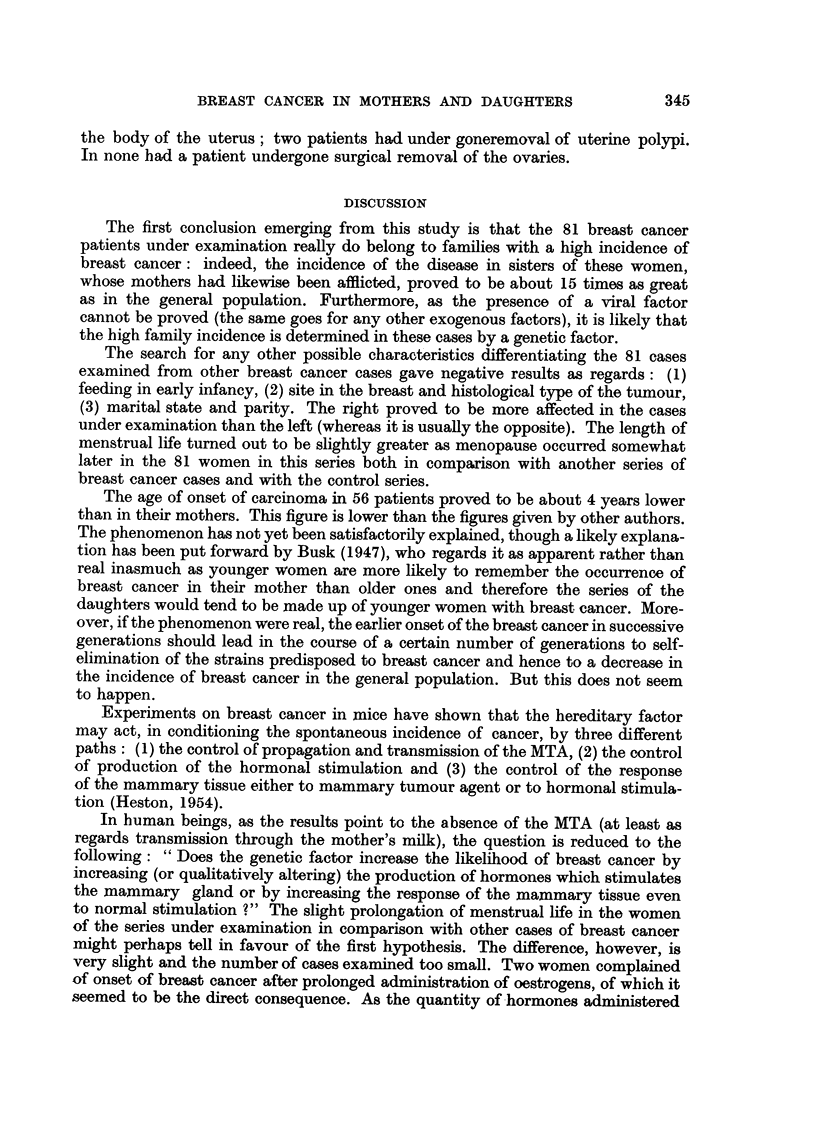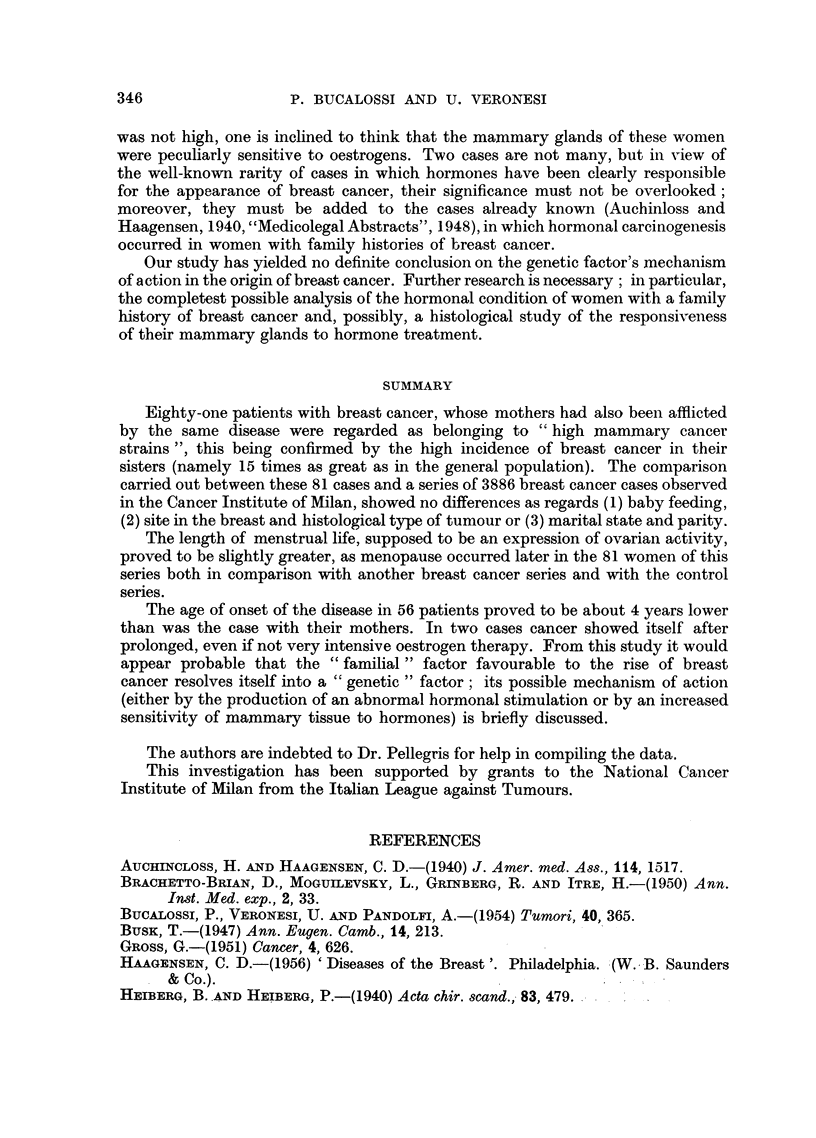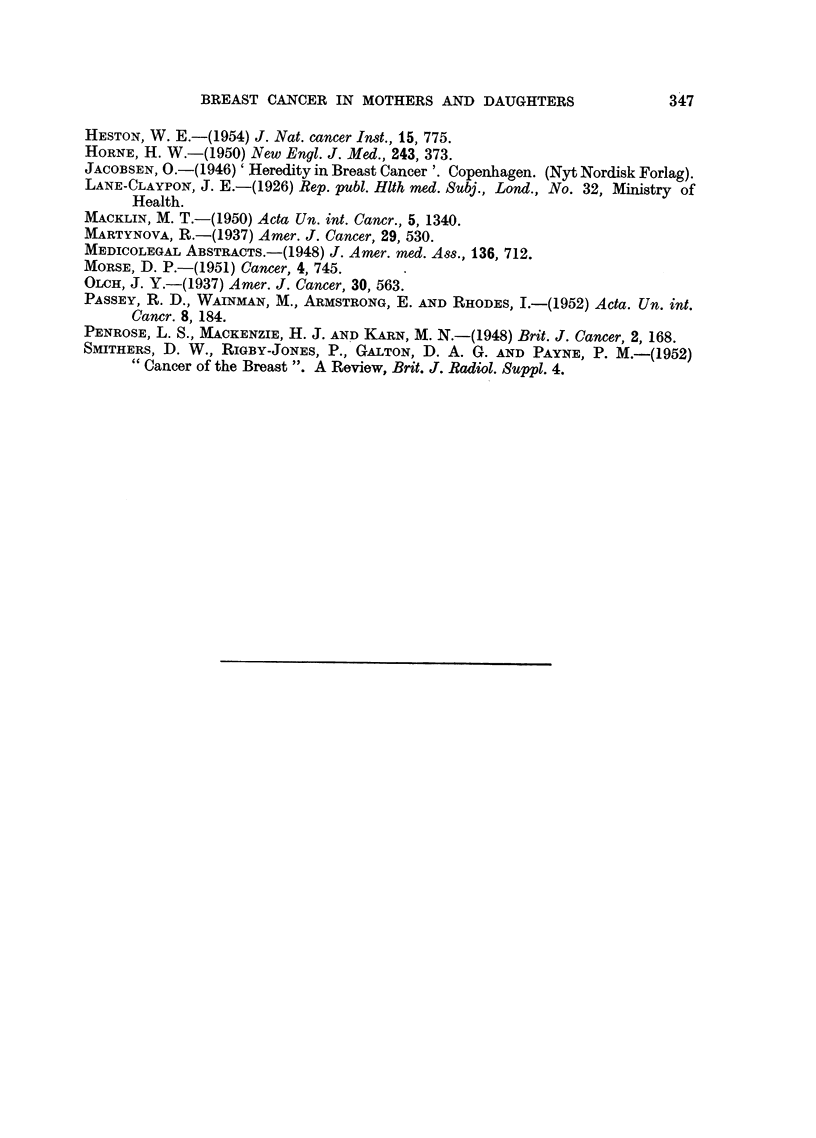# Some Observations on Cancer of the Breast in Mothers and Daughters

**DOI:** 10.1038/bjc.1957.41

**Published:** 1957-09

**Authors:** P. Bucalossi, U. Veronesi


### SOME OBSERVATIONS ON CANCER OF THE BREAST IN MOTHERS AND DAUGHTERS

P. BUCALOSSI AND U. VERONESI

*From the National Cancer Institute of Milan, Italy*

Received for publication July 13, 1957

EXPERIMENTAL studies on breast cancer have shown that for the tumour to develop in the mouse there are three essential factors : hormonal stimulation, the mammary tumour agent (MTA) and the genetic factor. The absence of any one of these three is sufficient to prevent or considerably lessen the incidence of the tumour. The environmental conditions constitute a fourth factor. A long series of researches has enabled us to determine with some exactness the part played by each of these factors, and to-day we can say that the aetiological factors in the mammary cancer of the mouse represent perhaps one of the surest points that have been reached among the many contradictions against which cancer research is struggling.

Things have a much more discouraging aspect in the human field. The information available from the literature on the aetiology of breast cancer is fragmentary and unconvincing. Of the influence of hormonal factors, studied in the main indirectly by analysis of the menstrual cycle and gynaecological disturbances, very little is known. A great deal of research has been done on the influence of hormones on the growth of cancer in the female breast, but this is not relevant to aetiology and is, anyway, a frequent source of paradoxical observations : witness the inhibiting action of oestrogens on breast cancer in elderly women and the beneficial action of orchidectomy and oestrogenic therapy in cancer of the male breast.

In the case of the hypothetical MTA in humans there has never been a sufficiently extensive survey to prove conclusively whether it exists or not.

The hereditary factor has been systematically studied by a large number of research workers (Lane-Claypon, 1926 ; Martynova, 1937 ; Jacobsen, 1946 ; Penrose, Mackenzie and Karn, 1948 ; Macklin, 1950 ; Passey *et al.*, 1952 ; Smithers *et al.*, 1952; Bucalossi, Veronesi and Pandolfi, 1954), but while the majority of them would admit the existence of a hereditary factor in the genesis of breast cancer in human beings, others (Passey *et al.*, 1952) deny this. The greatest obstacle to the study of the part played by the hereditary factor in human beings lies in the obvious impossibility of obtaining high incidence strains of breast cancer by means of appropriate crosses. However, cases of such crosses do arise by chance and hence we get families in which the incidence of breast cancer is particularly high.

In view of the very substantial breast cancer material at our disposal (3988 cases collected over 28 years), we thought it would be interesting to examine a series of cases in which a high family incidence of breast cancer pointed to a genetic predisposition to the disease. We might define these cases, in line with experimental usage, as belonging to "high mammary cancer strains" which, when sufficiently numerous to be significant statistically and when compared with other cases of breast cancer, constitute material suitable for multiple analysis. We give here the results obtained from the analysis of 81 cases of women with breast cancer whose mothers had died of or been affected with breast cancer.

### MATERIAL STUDIED

From the clinical case histories of 3988 breast cancer patients hospitalized in the Cancer Institute of Milan, between 1928 and 1956, it was noted that in 102 cases the patients were daughters of women affected with or who had died from cancer of the breast. Of these 102 cases it was possible to confirm definitely that 81 of the mothers had had breast cancer (by careful questioning of the relatives or by means of death certificates or hospital certificates) whereas in 21 cases this datum was uncertain and hence these were discarded. The 81 cases were carefully examined by analysis of the clinical case histories, questioning of relatives traced or of the patients themselves, if alive, and by means of numerous questionnaires sent to the families. The following points were examined with special attention : (*a*) incidence of breast cancer among sisters of the patients, (*b*) whether the patients had been breast-fed or artificially fed, (*c*) age of the mothers and daughters at the time of onset of breast cancer, (*d*) data regarding the site of the tumour and its histological type, (*e*) marital state, parity and menstrual history, (*f*) gynaecological precedents.

The control material consisted (1) of the remaining 3886 breast cancer cases hospitalized at the Institute from 1928 to 1956 and (2) of 1177 control patients without cancer hospitalized at the Institute during the same period. As these cases belonged to the same social classes and came from the same geographical areas and as the age distribution was sufficiently similar to that of the breast cancer cases, they seemed to us very suitable for the purpose.

### (a) *Incidence of breast cancer among sisters of the patients*

An obvious question which we asked ourselves on examining our 81 cases was whether the appearance of breast cancer in the mothers and daughters was to be considered as purely casual or whether a "familial" factor was involved. To answer this question, we sought information on other cases of breast cancer among the female relatives of the 81 patients on the hypothesis that if a "familial" factor exists, there would be a larger number of breast cancer cases among these relatives than in the population at large. Unfortunately, as most of the cases were hospitalized many years ago and many of them have died, it was impossible to reconstruct a satisfactory pedigree in the majority of the cases, and hence we limited ourselves to observing the incidence of breast cancer among the sisters of the patients, these being the most easily traceable relatives. It turned out that 10 patients had had one case of breast cancer among sisters and, in addition, one patient had had two cases ; altogether there were thus 12 cases of breast cancer among the sisters of the patients. Furthermore, one patient had lost a brother through breast cancer. The frequency of breast cancer among the sisters of our 81 patients worked out very high in comparison with the other statistics known to us, as seen in Table I. It is about 15 times higher than that to be found among the sisters of non-cancerous women and 3–4 times higher than that observed among the sisters of women with breast cancer.

TABLE I.—*Incidence of Breast Cancer among Sisters of Patients Suffering from the Disease, among Sisters of Controls and among Sisters of the Patients of Present-Series*

| Author | Number of cases | Number of breast cancer cases among sisters | Number of sisters with breast cancer every 100 cases |
|---|---|---|---|
| *Breast cancer cases :* | | | |
| Martynova, 1937 | 201 | 6 | 2·98 |
| Jacobsen, 1946 | 200 | 13 | 6·50 |
| Penrose *et al.*, 1948 | 510 | 23 | 4·51 |
| Smithers *et al.*, 1952 | 704 | 16 | 2·27 |
| Bucalossi *et al.*, 1954 | 230 | 12 | 5·22 |
| Haagensen, 1956 | 403 | 21 | 5·21 |
| *Control cases :* | | | |
| Jacobsen, 1946 | 200 | 2 | 1·00 |
| Bucalossi *et al.*, 1954 | 230 | 2 | 0·87 |
| *Present series* | 81 | 12 | 14·81 |

The high incidence of breast cancer among the sisters of the 81 cases under review confirms that the latter constitute a group of cases belonging to the high cancer strains in whose genesis a " familial " factor seems unquestionably to play a part.

(b) *Breast or artificial feeding of the patients as babies*

The problem of the possible existence of a viral factor which plays a similar part in the evolution of human breast cancer as that of the mammary tumour agent in the mouse has many times been put forward. Horne (1950) sent out a questionnaire to 88 mothers of women afflicted with breast cancer asking whether they had breast-fed their daughters or not. Of these 88, 9 replied that they had not, being unsuitable. On the basis of these replies Horne concluded that breast cancer can develop without the presence of any factor transmitted through the mother's milk. Gross (1951) commenting on these results, pointed out that it was difficult for an elderly woman to remember whether 30 or 40 years ago she had not breast-fed her daughter, if only for a few minutes. Further, even admitting that in fact the 9 cases had received no milk from the mother, this does not rule out the possibility that the remaining 79 cases were contaminated at least to some extent by this means. Penrose, Mackenzie and Karn (1948), comparing a series of 79 breast cancer cases with a " familial " breast cancer history with another series of 360 unselected breast cancer cases, found that in the former series 93 per cent had been breast-fed as against 87 per cent in the second series.

In 13 out of the 81 cases under review we did not manage to get information about feeding. Of the remaining 68 patients 56 had been breast-fed, 9 had been foster-fed and 3 artificially fed. These results are compared in Table II with 3886 cases of breast cancer observed in our Institute and with 1177 patients not afflicted with tumours also hospitalized at our Institute. It will be noted that there are no significant differences between the three groups of cases. Thus this result is not favourable to the hypotheses of transmission of a supposed " tumoral agent " through the mother's milk.

TABLE II.—*Type of Baby Feeding in* 81 *Patients of Present Series, in* 3886 *Breast Cancer Patients and* 1177 *Control Patients*

| Type of baby feeding | Present series cases No. | Present series cases %* | Breast cancer cases No. | Breast cancer cases %* | Control cases No. | Control cases %* |
|---|---|---|---|---|---|---|
| Breast-nursed by mother | 56 | 82·4 | 2812 | 84·52 | 805 | 84·21 |
| Foster-nursed | 9 | 13·2 | 417 | 12·53 | 120 | 12·55 |
| Artificially fed | 3 | 4·4 | 98 | 2·95 | 31 | 3·24 |
| Total of known cases | 68 | 100·0 | 3327 | 100·00 | 956 | 100·00 |
| Unknown cases | 13 | — | 559 | — | 221 | — |
| Total | 81 | | 3886 | | 1177 | |

* The percentage is referred to the total number of cases in which the type of lactation was known.

(c) *Ages of mothers and daughters at the onset of breast cancer*

In 1951 Morse reported a series of cases he had noted of breast cancer which had appeared in mothers and daughters, observing a greater precocity in the appearance of cancer in the daughters by over 10 years. This result agreed with the one drawn from 21 cases of Jacobsen's (1946) monograph in which the difference worked out at 9 years. Haagensen (1956) in his recent treatise reports a series of 28 cases in which both mother and daughter had died of breast cancer. The average age of the mothers was 57·11 years and that of the daughters 46·21 years, giving a difference of 10·90 years.

In 58 of our cases it was possible to know the exact age of the mothers at the onset of breast cancer. Table III shows the 58 cases with the ages of mothers and daughters. We give the data in full in case they should be of use to anyone wishing to utilize them in conjunction with other case material for statistical purposes. The average age of the 58 patients at onset was 49·48 years, the average age of the mothers 53·66 years. The difference of 4·17 years is thus far lower than that given by previous writers (Table IV).

(d) *Site and histological type of the tumours*

Of the 81 cases 42 were located in the right breast, 36 in the left and 3 were bilateral. In 59 cases the exact site of the neoplasia was known : in 22 the tumour was situated in the external upper quadrant, in 11 in the internal upper quadrant, in 4 cases in the external lower quadrant, in 5 in the internal lower quadrant and in 10 cases in the central quadrant. In 2 cases the neoplasm occupied the upper half and in one the lower half, in one case the left half and in 3 cases a large part of the mammary gland. The distribution by quadrants thus does not differ appreciably from the usual run of breast cancers, as shown in Table V where the 59 cases are compared with a series of 1000 consecutive cases of breast cancer taken from the 3886 of the Cancer Institute of Milan.

Of the 81 cases of the present series 15 had previously been operated on at other hospitals, and we were able to obtain the slides for consultation in 7 of these,

TABLE III.—*Age, at Onset, of* 58 *Mothers and Daughters with Breast Cancer*

| Record number | Mother's age | Daughter's age | Difference |
|---|---|---|---|
| 12 | 72 | 47 | −25 |
| 26 | 49 | 37 | −12 |
| 182 | 46 | 41 | −5 |
| 430 | 37 | 35 | −2 |
| 671 | 67 | 49 | −18 |
| 688 | 62 | 54 | −8 |
| 798 | 44 | 40 | −4 |
| 954 | 51 | 50 | −1 |
| 986 | 60 | 65 | +5 |
| 1065 | 39 | 53 | +14 |
| 1126 | 65 | 48 | −17 |
| 1271 | 36 | 31 | −5 |
| 1376 | 52 | 50 | −2 |
| 1488 | 63 | 55 | −8 |
| 1577 | 61 | 49 | −12 |
| 1617 | 73 | 56 | −17 |
| 1629 | 70 | 56 | −14 |
| 1657 | 72 | 55 | −17 |
| 1661 | 51 | 49 | −2 |
| 1873 | 48 | 44 | −4 |
| 2171 | 48 | 45 | −3 |
| 2238 | 40 | 54 | +14 |
| 2264 | 43 | 52 | +9 |
| 2347 | 67 | 52 | −15 |
| 2376 | 50 | 45 | −5 |
| 2395 | 48 | 65 | +17 |
| 2413 | 44 | 39 | −5 |
| 2418 | 33 | 48 | +15 |
| 2432 | 66 | 47 | −19 |
| 2439 | 38 | 36 | −2 |
| 2544 | 44 | 53 | +9 |
| 2686 | 46 | 27 | −19 |
| 2739 | 57 | 40 | −17 |
| 2757 | 51 | 36 | −15 |
| 2779 | 56 | 51 | −5 |
| 2785 | 51 | 69 | +18 |
| 2926 | 50 | 43 | −7 |
| 2934 | 45 | 34 | −11 |
| 3001 | 60 | 65 | +5 |
| 3078 | 44 | 49 | +5 |
| 3092 | 66 | 62 | −4 |
| 3123 | 57 | 42 | −15 |
| 3216 | 34 | 57 | +23 |
| 3283 | 44 | 70 | +26 |
| 3399 | 68 | 72 | +4 |
| 3413 | 35 | 62 | +27 |
| 3476 | 46 | 60 | +14 |
| 3498 | 72 | 46 | −26 |
| 3518 | 70 | 78 | +8 |
| 3530 | 59 | 54 | −5 |
| 3532 | 60 | 61 | +1 |
| 3535 | 76 | 44 | −32 |
| 3680 | 43 | 42 | −1 |
| 3733 | 62 | 43 | −19 |
| 3778 | 48 | 31 | −17 |
| 3823 | 67 | 54 | −13 |
| 3871 | 40 | 44 | +4 |
| 3876 | 66 | 34 | −32 |
| Average | 53·655 | 49·482 | −4·173 |

TABLE IV.—*Average Age of Breast Cancer, at Onset, in Mothers and Daughters, in Jacobsen's, Morse's, Haagensen's and Present Series*

| Author | Number of cases | Mean age of mothers | Mean age of daughters | Difference |
|---|---|---|---|---|
| Jacobsen, 1946 | 21 | 57·38 | 48·38 | 9·00 |
| Morse, 1951 | 13 | 57·46 | 47·00 | 10·46 |
| Haagensen, 1956 | 28 | 57·11 | 46·21 | 10·90 |
| Present series | 58 | 53·66 | 49·48 | 4·17 |
| Total | 120 | 55·52 | 48·26 | 7·26 |

TABLE V.—*Site of Breast Cancer in* 59 *Cases of the Present Series and in* 1000 *Consecutive Breast Cancer Cases of the National Cancer Institute of Milan*

| Quadrant | 59 cases of present series | | 1000 consecutive cases of breast cancer | |
|---|---|---|---|---|
| | No. | % | No. | % |
| Outer upper | 22 | 37·3 | 383 | 38·3 |
| Inner upper | 11 | 18·6 | 142 | 14·2 |
| Outer lower | 4 | 6·8 | 97 | 9·7 |
| Inner lower | 5 | 8·5 | 33 | 3·3 |
| Central | 10 | 16·9 | 156 | 15·6 |
| More than one quadrant | 7 | 11·9 | 189 | 18·9 |
| Total | 59 | 100·0 | 1000 | 100·0 |

it not being possible in the other 8 cases. Altogether, therefore, we possess the histological examinations of 73 patients, divided as follows :

| | |
|---|---|
| Carcinoma of no special type | 67 |
| Infiltrating comedocarcinoma | 3 |
| Papilliferous carcinoma | 2 |
| Apocrine carcinoma | 1 |

In 2 cases we found carcinoma coexisting with chronic cystic mastitis. In one case of papilliferous carcinoma the mammary gland proved to be disseminated with intraductal papillomata. Excluding the three cases of bilateral carcinoma the contralateral breast proved to have normal clinical characteristics in all cases with the exception of one patient of 50 years in whom chronic cystic mastitis was found. In another case, that of a woman of 40 years, a fibro-adenoma had been removed 17 years before. Three patients had suffered from mastitis during breast-feeding of their children.

Two patients had been subjected to prolonged hormonal treatment in the years prior to the appearance of breast cancer. In the first case, a woman of 50 years had undergone treatment with oestrogen hormones continuously for two years previous to the appearance of cancer, having taken a total dose of 50 mg. of estradiol benzoate. In the second case, a woman suffering from mastodinia began to have diethyl-stilboestrol hormone treatment at the age of 43 years and continued this on and off for four years, the total dose being about 800 mg. of hormone. After this period the patient noticed a hardening of the breast and a biopsy revealed the existence of widespread cystic mastitis with numerous minute nests of multicentric carcinoma.

(e) *Marital state and menstrual history*

Of the 81 patients 14 were single and 8 married without children : the nulliparous therefore numbered 22 ; 13 patients had one child, 33 two or three children and 13 had four or more. Table VI gives a comparison of the 81 cases as regards parity with 3833 patients suffering from breast cancer, comparable as regards the above classification. Table VII compares the average number of children per patient in the present series with our breast cancer cases generally and with the controls. These data confirmed the well-known fact that the incidence of human breast cancer is in inverse proportion to the number of pregnancies.

TABLE VI.—*Parity in* 81 *Cases of Present Series and in* 3733* *Breast Cancer Cases of the National Cancer Institute of Milan*

| | 81 case of present series | | 3733 breast cancer cases | |
|---|---|---|---|---|
| Parity | No. | % | No. | % |
| Nulliparous . . . . | 22 | 27·16 | 932 | 24·96 |
| With 1 child . . . | 13 | 16·05 | 730 | 19·56 |
| With 2 or 3 children . . | 33 | 40·74 | 1227 | 32·87 |
| With 4 or more children . | 13 | 16·05 | 844 | 22·61 |
| Total . | 81 | 100·00 | 3733 | 100·00 |

* Among the 3886 cases of breast cancer, in 153 the parity was unknown.

TABLE VII.—*Average Number of Children in Cases of Present Series, in Breast Cancer Cases and in Control Cases*

| | Average number of children |
|---|---|
| 81 cases of present series . . | 2·16 |
| 3733 breast cancer cases . . | 2·26 |
| 1060 control cases. . . . | 2·82 |

The length of menstrual history, which can be taken as an expression of ovarian activity, has been investigated in several series of women with breast cancer (Lane-Claypon, 1926 ; Olch, 1937 ; Heiberg and Heiberg, 1940 ; Brachetto-Brian *et al.*, 1950 ; Smithers *et al.*, 1952) and the investigators agree in finding longer menstrual life in women with breast cancer than in the general population. In some of these studies, however, the control material is insufficiently convincing. Indeed, for a comparison to be valid, both the patients and the controls should belong strictly to the same social class and to the same geographical area (it is enough to remember the difference between women from rural and urban areas or from different altitudes) and, if possible, comparable as regards parity. Bearing these factors in mind, we examined and compared the age of menarche and of menopause in the 81 cases of the present series, in 1000 cases of breast cancer and in 500 non-cancerous women used for control purposes. All those cases were collected in the Cancer Institute of Milan during the same period of time (1928–56) and represent a preliminary series of cases from a wider investigation which will be published later on. Results are given in Tables VIII and IX. While the average ages of onset of menstruation show no significant differences as between the 81 cases of the present series, the 1000 cases of breast cancer and the 500 non-cancerous controls, the average age of menopause in the 46 cases in which menstrual life had ceased (48·85 years) is about a year later than the average age of menopause among the controls (47·84) and about 9 months later than the average age of menopause in the 1000 breast cancer cases (48·09).

TABLE VIII.—*Age Distribution at the Menarche of the* 81 *Patients of Present Series, of* 1000 *Patients with Cancer of the Breast and* 500 *Control Patients*

| Age at menarche (years) | Present series cases No. | Present series cases % | Breast cancer cases No. | Breast cancer cases % | Controls No. | Controls % |
|---|---|---|---|---|---|---|
| 9 | 0 | 0·0 | 6 | 0·6 | 2 | 0·4 |
| 10 | 1 | 1·2 | 18 | 1·8 | 7 | 1·4 |
| 11 | 8 | 9·9 | 103 | 10·3 | 42 | 8·4 |
| 12 | 11 | 13·6 | 168 | 16·8 | 67 | 13·4 |
| 13 | 15 | 18·5 | 200 | 20·0 | 122 | 24·4 |
| 14 | 22 | 27·2 | 197 | 19·7 | 105 | 21·0 |
| 15 | 14 | 17·3 | 146 | 14·6 | 65 | 13·0 |
| 16 | 8 | 9·9 | 90 | 9·0 | 53 | 10·6 |
| 17 | 0 | 0·0 | 47 | 4·7 | 15 | 3·0 |
| 18 | 2 | 2·4 | 19 | 1·9 | 15 | 3·0 |
| 19 | 0 | 0·0 | 1 | 0·1 | 4 | 0·8 |
| 20 | 0 | 0·0 | 5 | 0·5 | 3 | 0·6 |
| Total | 81 | 100·0 | 1000 | 100·0 | 500 | 100·0 |
| | Mean age : 13·654 years | | Mean age : 13·631 years | | Mean age : 13·788 years | |

TABLE IX.—*Age Distribution at the Menopause of* 46* *Patients of the Present Series, of* 1000 *Patients with Cancer of the Breast and of* 500 *Control Patients*

| Age at menopause (years) | Present series cases* No. | Present series cases* % | Breast cancer cases No. | Breast cancer cases % | Control cases No. | Control cases % |
|---|---|---|---|---|---|---|
| Less than 38 | 0 | 0·0 | 13 | 1·3 | 12 | 2·4 |
| 38–40 | 0 | 0·0 | 65 | 6·5 | 22 | 4·4 |
| 41–43 | 5 | 10·9 | 82 | 8·2 | 42 | 8·4 |
| 44–46 | 6 | 13·0 | 151 | 15·1 | 97 | 19·4 |
| 47–49 | 13 | 28·3 | 232 | 23·2 | 122 | 24·4 |
| 50–52 | 14 | 30·4 | 319 | 31·9 | 149 | 29·8 |
| 53–55 | 8 | 17·4 | 109 | 10·9 | 45 | 9·0 |
| More than 55 | 0 | 0·0 | 29 | 2·9 | 11 | 2·2 |
| Total | 46 | 100·0 | 1000 | 100·0 | 500 | 100·0 |
| | Mean age : 48·848 years | | Mean age : 48·093 years | | Mean age : 47·838 years | |

* Out of 81 patients, 35 had not reached the menopause.

### (f) *Previous gynaecological diseases*

Of the 81 patients 74 had not suffered from gynaecological complaints ; 4 patients had been operated on for or were afflicted with uterine myoma ; one patient of 72 years had been treated with radium 10 years before for adenocarcinoma of the body of the uterus ; two patients had under goneremoval of uterine polypi. In none had a patient undergone surgical removal of the ovaries.

### DISCUSSION

The first conclusion emerging from this study is that the 81 breast cancer patients under examination really do belong to families with a high incidence of breast cancer : indeed, the incidence of the disease in sisters of these women, whose mothers had likewise been afflicted, proved to be about 15 times as great as in the general population. Furthermore, as the presence of a viral factor cannot be proved (the same goes for any other exogenous factors), it is likely that the high family incidence is determined in these cases by a genetic factor.

The search for any other possible characteristics differentiating the 81 cases examined from other breast cancer cases gave negative results as regards : (1) feeding in early infancy, (2) site in the breast and histological type of the tumour, (3) marital state and parity. The right proved to be more affected in the cases under examination than the left (whereas it is usually the opposite). The length of menstrual life turned out to be slightly greater as menopause occurred somewhat later in the 81 women in this series both in comparison with another series of breast cancer cases and with the control series.

The age of onset of carcinoma in 56 patients proved to be about 4 years lower than in their mothers. This figure is lower than the figures given by other authors. The phenomenon has not yet been satisfactorily explained, though a likely explanation has been put forward by Busk (1947), who regards it as apparent rather than real inasmuch as younger women are more likely to remember the occurrence of breast cancer in their mother than older ones and therefore the series of the daughters would tend to be made up of younger women with breast cancer. Moreover, if the phenomenon were real, the earlier onset of the breast cancer in successive generations should lead in the course of a certain number of generations to self-elimination of the strains predisposed to breast cancer and hence to a decrease in the incidence of breast cancer in the general population. But this does not seem to happen.

Experiments on breast cancer in mice have shown that the hereditary factor may act, in conditioning the spontaneous incidence of cancer, by three different paths : (1) the control of propagation and transmission of the MTA, (2) the control of production of the hormonal stimulation and (3) the control of the response of the mammary tissue either to mammary tumour agent or to hormonal stimulation (Heston, 1954).

In human beings, as the results point to the absence of the MTA (at least as regards transmission through the mother's milk), the question is reduced to the following : " Does the genetic factor increase the likelihood of breast cancer by increasing (or qualitatively altering) the production of hormones which stimulates the mammary gland or by increasing the response of the mammary tissue even to normal stimulation ?" The slight prolongation of menstrual life in the women of the series under examination in comparison with other cases of breast cancer might perhaps tell in favour of the first hypothesis. The difference, however, is very slight and the number of cases examined too small. Two women complained of onset of breast cancer after prolonged administration of oestrogens, of which it seemed to be the direct consequence. As the quantity of hormones administered was not high, one is inclined to think that the mammary glands of these women were peculiarly sensitive to oestrogens. Two cases are not many, but in view of the well-known rarity of cases in which hormones have been clearly responsible for the appearance of breast cancer, their significance must not be overlooked; moreover, they must be added to the cases already known (Auchinloss and Haagensen, 1940, "Medicolegal Abstracts", 1948), in which hormonal carcinogenesis occurred in women with family histories of breast cancer.

Our study has yielded no definite conclusion on the genetic factor's mechanism of action in the origin of breast cancer. Further research is necessary; in particular, the completest possible analysis of the hormonal condition of women with a family history of breast cancer and, possibly, a histological study of the responsiveness of their mammary glands to hormone treatment.

### SUMMARY

Eighty-one patients with breast cancer, whose mothers had also been afflicted by the same disease were regarded as belonging to "high mammary cancer strains", this being confirmed by the high incidence of breast cancer in their sisters (namely 15 times as great as in the general population). The comparison carried out between these 81 cases and a series of 3886 breast cancer cases observed in the Cancer Institute of Milan, showed no differences as regards (1) baby feeding, (2) site in the breast and histological type of tumour or (3) marital state and parity.

The length of menstrual life, supposed to be an expression of ovarian activity, proved to be slightly greater, as menopause occurred later in the 81 women of this series both in comparison with another breast cancer series and with the control series.

The age of onset of the disease in 56 patients proved to be about 4 years lower than was the case with their mothers. In two cases cancer showed itself after prolonged, even if not very intensive oestrogen therapy. From this study it would appear probable that the "familial" factor favourable to the rise of breast cancer resolves itself into a "genetic" factor; its possible mechanism of action (either by the production of an abnormal hormonal stimulation or by an increased sensitivity of mammary tissue to hormones) is briefly discussed.

The authors are indebted to Dr. Pellegris for help in compiling the data.

This investigation has been supported by grants to the National Cancer Institute of Milan from the Italian League against Tumours.

### REFERENCES

AUCHINCLOSS, H. AND HAAGENSEN, C. D.—(1940) *J. Amer. med. Ass.*, **114**, 1517.

BRACHETTO-BRIAN, D., MOGUILEVSKY, L., GRINBERG, R. AND ITRE, H.—(1950) *Ann. Inst. Med. exp.*, **2**, 33.

BUCALOSSI, P., VERONESI, U. AND PANDOLFI, A.—(1954) *Tumori*, **40**, 365.

BUSK, T.—(1947) *Ann. Eugen. Camb.*, **14**, 213.

GROSS, G.—(1951) *Cancer*, **4**, 626.

HAAGENSEN, C. D.—(1956) 'Diseases of the Breast'. Philadelphia. (W. B. Saunders & Co.).

HEIBERG, B. AND HEIBERG, P.—(1940) *Acta chir. scand.*, **83**, 479.

HESTON, W. E.—(1954) *J. Nat. cancer Inst.*, **15**, 775.
HORNE, H. W.—(1950) *New Engl. J. Med.*, **243**, 373.
JACOBSEN, O.—(1946) ' Heredity in Breast Cancer '. Copenhagen. (Nyt Nordisk Forlag).
LANE-CLAYPON, J. E.—(1926) *Rep. publ. Hlth med. Subj., Lond., No.* 32, Ministry of Health.
MACKLIN, M. T.—(1950) *Acta Un. int. Cancr.*, **5**, 1340.
MARTYNOVA, R.—(1937) *Amer. J. Cancer*, **29**, 530.
MEDICOLEGAL ABSTRACTS.—(1948) *J. Amer. med. Ass.*, **136**, 712.
MORSE, D. P.—(1951) *Cancer*, **4**, 745.
OLCH, J. Y.—(1937) *Amer. J. Cancer*, **30**, 563.
PASSEY, R. D., WAINMAN, M., ARMSTRONG, E. AND RHODES, I.—(1952) *Acta. Un. int. Cancr.* **8**, 184.
PENROSE, L. S., MACKENZIE, H. J. AND KARN, M. N.—(1948) *Brit. J. Cancer*, **2**, 168.
SMITHERS, D. W., RIGBY-JONES, P., GALTON, D. A. G. AND PAYNE, P. M.—(1952) " Cancer of the Breast ". A Review, *Brit. J. Radiol. Suppl.* 4.